# Optimizing Dynamic White Lighting for practical application

**DOI:** 10.1002/alz.094243

**Published:** 2025-01-09

**Authors:** MD. Azaharuddin Ansari, Jonathon David White

**Affiliations:** ^1^ Yuan Ze University, Taoyuan CIty, Taoyuan Taiwan

## Abstract

**Background:**

Effect of dynamic lighting on sleep were studied since 1980’s. Traditional light sources were used due to lack of advancement in technology and also researchers assumed illuminance as cause of melatonin suppression. This led researchers to use high illuminance to suppress melatonin at day time. Later, it was discovered that intrinsic photoreceptor retinal ganglion cell (ipRGC), sensitive to specific wavelength, has direct impact on the level of melatonin, that shifted the attention to the actual wavelength of the stimulating light. With the invention of LEDs, having various emission profiles has allowed researcher to propose numerous LED using 5 to 8 types of LED for appropriate delivery of dynamic light to control melatonin secretion whole day. Better lighting has been shown to improve sleep, reduce anxiety/depression, and improve mood in dementia patients.

**Method:**

A C‐program was developed to calculate various lighting parameters of combined spectra of LED strips. Brute force optimization was used to calculate the power to be supplied to each of the input LED strips to obtain a combined white light for dawn, day and dusk based on the required visual (e.g. CRI>90, Duv < 0.001) and non‐visual parameters for minimum power consumption and lowest material cost.

**Result:**

We investigated 3 red, green, blue, ice blue, and warm white (R‐G‐B‐IB‐WW) systems from different manufactures. Only in one system, did the blue LED allow increased efficiency (<1%), demonstrating that a 4 LED system (R‐G‐IB‐WW) is sufficient i.e. blue LED is unnecessary. We added a monochromatic Cyan LED strip, its spectra overlap the action spectra of ipRGC, hoping it could help to achieve better stimulation of ipRGC cells with lower power consumption. While m‐EDI increased, but m‐EDI/W input power did not. Cyan is thus unnecessary.

**Conclusion:**

We have shown that an LED system with only 4 types of LEDS (2 monochrome and 2 broadband) can generate dawn to dusk dynamic white light suitable for both visual and non‐visual needs of dementia patients (as widely accepted by researchers over the world) with high electrical to optical efficiency. This approach, using fewer LED strips and lower energy consumption, is more financially appealing.

